# Glycerol-3-phosphate acyltransferase 4 is essential for the normal development of reproductive organs and the embryo in *Brassica napus*

**DOI:** 10.1093/jxb/eru199

**Published:** 2014-05-12

**Authors:** Xue Chen, Guanqun Chen, Martin Truksa, Crystal L. Snyder, Saleh Shah, Randall J. Weselake

**Affiliations:** ^1^Agricultural Lipid Biotechnology Program, Department of Agricultural, Food and Nutritional Science, University of Alberta, Edmonton, Alberta, Canada T6G 2P5; ^2^Plant Biotechnology, Alberta Innovates-Technology Futures, Vegreville, Alberta, Canada T9C 1T4

**Keywords:** *Brassica napus*, cutin biosynthesis, embryo development, female fertility, GPAT, reproductive organ.

## Abstract

The enzyme *sn*-glycerol-3-phosphate acyltransferase 4 (GPAT4) is involved in the biosynthesis of plant lipid poly-esters. The present study further characterizes the enzymatic activities of three endoplasmic reticulum-bound GPAT4 isoforms of *Brassica napus* and examines their roles in the development of reproductive organs and the embryo. All three BnGPAT4 isoforms exhibited *sn*-2 acyltransferase and phosphatase activities with dicarboxylic acid-CoA as acyl donor. When non-substituted acyl-CoA was used as acyl donor, the rate of acylation was considerably lower and phosphatase activity was not manifested. RNA interference (RNAi)-mediated down-regulation of all *GPAT4* homologues in *B. napus* under the control of the napin promoter caused abnormal development of several reproductive organs and reduced seed set. Microscopic examination and reciprocal crosses revealed that both pollen grains and developing embryo sacs of the *B. napus gpat4* lines were affected. The *gpat4* mature embryos showed decreased cutin content and altered monomer composition. The defective embryo development further affected the oil body morphology, oil content, and fatty acid composition in *gpat4* seeds. These results suggest that GPAT4 has a critical role in the development of reproductive organs and the seed of *B. napus*.

## Summary

This study revealed critical physiological roles for BnGPAT4s in reproductive organ and embryo development. This information adds further to knowledge on the physiological roles of these multifunctional enzymes in plant development.

## Introduction

The *sn*-glycerol-3-phosphate acyltransferase (GPAT) family is involved in acyl-lipid biosynthesis and plays a pivotal role in plant development (Chen *et al.*, 2011*a*). GPAT catalyses the transfer of a fatty acid moiety from acyl-coenzyme A (acyl-CoA) or acyl-acyl carrier protein (acyl-ACP) to the *sn*-1 or 2 position of *sn*-glycerol 3-phosphate (G3P) forming lysophosphatidic acid (LPA). Additionally, certain GPATs also possess phosphatase activity and thus result in the formation of monoacylglycerols (MAGs) (Yang *et al.*, 2012). Based on subcellular localization, GPATs can be categorized into three types: (i) the plastidial soluble GPATs; (ii) the mitochondrial membrane-bound GPATs; and (iii) the endoplasmic reticulum (ER) membrane-bound GPATs. The plastidial GPAT (also annotated as ATS1) was first cloned from squash (Ishizaki *et al.*, 1988). Its homologues have also been cloned and characterized from several plant species including pea (*Pisum sativum*) and *Arabidopsis* (Weber *et al.*, 1991; Nishida *et al.*, 1993; Xu *et al.*, 2006). Plastidial GPATs were shown to catalyse only the *sn*-1 acylation of G3P and are primarily involved in the prokaryotic glycerolipid biosynthesis pathway. Three isoforms encoding the mitochondrial membrane-bound GPATs (i.e. GPATs 1–3) have been cloned in *Arabidopsis* (Zheng *et al.*, 2003). Among them, AtGPAT1 was essential for tapetal differentiation and male fertility, and *Arabidopsis gpat1* T-DNA mutants had altered fatty acid compositions in the storage and membrane lipids of flower buds, pollen grains, and seeds (Zheng *et al.*, 2003). Recently, AtGPAT1 was shown to possess only *sn*-2 acyltransferase activity with acyl substrate preference for mono behenoyl (22:0)-CoA and mono 22:0 α,ω-dicarboxylic acid (DCA)-CoA (Yang *et al.*, 2012). There are six members of the ER-bound GPATs in *Arabidopsis* (i.e. AtGPAT4–AtGPAT9 (Zheng *et al.*, 2003; Li *et al.*, 2007; Gidda *et al.*, 2009). AtGPAT4, 5, 6, and 8 were found to be involved in the synthesis of extracellular lipid polyesters (i.e. cutin and suberin) by providing acylglycerol to the polyester matrices (Beisson *et al.*, 2007; Li *et al.*, 2007; Li-Beisson *et al.*, 2009). In *in vitro* enzyme assays, AtGPAT4, 6, and 8 were shown to be bi-functional for acyltransferase (mainly at the *sn*-2 position) and phosphatase activities resulting in *sn*-2-MAG formation. In comparison, AtGPAT5 and 7 only exhibited strong preference for *sn*-2 acylation without phosphatase activity (Yang *et al.*, 2012). Although the physiological and catalytic functions of AtGPAT9 are still unclear, it was predicted to be involved in membrane and storage lipid biosynthesis (Gidda *et al.*, 2009). Interestingly, GPAT6 was shown to play a role in the development of the tapetum ER profile and pollen grains (Li *et al.*, 2012), suggesting that in addition to extracellular lipid polyester biosynthesis, the ER-bound GPATs may have other unknown physiological functions.

As a close relative of *Arabidopsis*, *Brasscia napus* is an important oilseed crop with a complex polyploid genetic background. Previously, three homologues of *GPAT4* were cloned in *B. napus* and their important roles in cutin accumulation were revealed, via an RNA interference (RNAi) approach to down-regulate *GPAT4* expression constitutively in vegetative tissues [under the direction of the *Cauliflower mosaic virus* (CaMV) 35S promoter] (Chen *et al.*, 2011*b*). The present study further investigates the enzymatic properties of the three GPAT4 isoforms and the functions of the isoforms in the development of reproductive organs and embryos of *B. napus*. The expression profiles of *B. napus* genes encoding several mitochondrial and ER-bound GPATs were investigated using quantitative real-time PCR (qRT-PCR). *GPAT4* exhibited the highest expression level among all investigated *GPAT* genes in maturing embryos. All three BnGPAT4 isoforms showed *sn*-2 acyltransferase and phosphatase activities when DCA-CoA was used as acyl donor. Acylation rates, however, were considerably lower with non-substituted acyl-CoA but, under these conditions, phosphatase activity was not manifested. With an RNAi silencing approach, under the direction of a napin promoter, abnormal development of the inflorescence, reduced seed set, decreased embryonic cutin and seed oil content, and an altered fatty acid composition were observed in the *B. napus gpat4* lines. In addition, examination of pollen and the embryo sac, and reciprocal crosses, suggested that down-regulation of *GPAT4* caused impaired female fertility, which is the main reason for reduced seed set. The results strongly suggest that in addition to cutin synthesis, GPAT4 plays important physiological roles in reproductive organ and embryo development in *B. napus*.

## Materials and methods

### Plant growth

*Brassica napus* double haploid line (DH12075) plants were grown in a growth chamber at 23 °C under an 18h day/6h night cycle.

### Synthesis of 16:0 DCA-CoA and 16:0 DCA-MAGs

The substrate 16:0 DCA-CoA was synthesized according to Kawaguchi *et al.* (1981) using 16:0 DCA, carbonyldiimidazole, and tetrahydrofuran. Briefly, 5 μmol of 16:0 DCA and 6 μmol of carbonyldiimidazole were mixed in 0.2ml of tetrahydrofuran. The mixture was filled with N_2_, sealed, and rapidly stirred at 1000rpm under room temperature for 30min. After the solvent had been evaporated under N_2_, the residue was dissolved in 0.2ml of tetrahydrofuran/H_2_O (2:1, v/v) and 5 μmol of freshly prepared CoASH was added, with the final pH being adjusted to 7.0–7.5. The product 16:0 DCA-CoA was precipitated by 7% perchloric acid, and the remaining 16:0-DCA was extracted by hexane. The 16:0 DCA-CoA precipitate was washed with 0.7% perchloric acid and dissolved in 0.01M NaOAc/EtOH (1:1, v/v; pH 5.2), filled with N_2_, and stored at –20 °C (Yang *et al.*, 2010). The purity of the 16:0 DCA-CoA was checked by thin-layer chromatography (TLC) with butanol:water:acetic acid (5:3:2, v/v/v) as the developing solvent and the concentration was determined by gas chromatography/mass spectrometry (GC/MS) with internal standard (17:0 methyl ester) after methylation with 2% sulphuric acid in methanol.

The *sn*-1 and *sn*-2 16:0 DCA-MAGs were synthesized according to Yang *et al.* (2010). For the synthesis of *sn*-2 16:0 DCA-MAG, a mixture containing 1ml of tetrahydrofuran, 9 μmol of 4-(dimethylamino) pyridine, 18 μmol of *N*-(3-dimethylaminopropyl)-*N*-ethylcarbodiimide hydrochloride, 27 μmol of 1,3-benzylidene, and 18 μmol of 16:0-DCA was stirred at room temperature for 24h. Subsequently, the reaction mixture was diluted with 5ml of ethyl acetate and washed with 3ml of water and 3ml of saturated NaCl solution. The organic layer was extracted and dried under N_2_. To remove the benzylidene group, 1–4ml of trimethyl borate and 0.0015g of boric acid powder were added to the mixture, which was stirred at 98 °C for 20min, dried under N_2_, and heated at 98 °C for another 10min. The product was extracted with 5ml of diethyl ether and washed three times with 2ml of water. The organic extract was applied to boric acid-TLC in a developing solvent of chloroform:acetone (1:1, v/v) with regular 16:0-MAG as standard. The 16:0-DCA *sn*-2 MAG was eluted from the boric acid–silica with chloroform:acetone (1:1, v/v), washed with ice-cold water, and dried down under N_2_ (Yang *et al.*, 2010). The procedure for synthesis of 16:0 DCA *sn*-1 MAG was similar, except that (*R*) (–)-2,2-dimethyl-1,3-dioxolane-4-methanol was used to replace 1,3-benzylidene.

### Heterologous expression of BnGPAT4s in the yeast *gat1Δ* strain and enzyme assays

BnGPAT4s were produced in yeast strain *gat1Δ* for *in vitro* enzyme assay (Chen *et al.*, 2011*b*). The GPAT enzyme assay was performed according to the method reported in Yang *et al.* (2010). A 20 μg aliquot of yeast microsomal protein was incubated with 0.5mM [^14^C(U)]G3P (0.1 μCi) and 45 μM acyl-CoA in a buffer containing 37.5mM Tris-HCl (pH 7.5), 2mM MgCl_2_, 4mM NaF, 1mM dithiothreitol (DTT), and 0.1% bovine serum albumin (BSA; w/v) at room temperature for 10min. The reactions were quenched with 5 μl of acetonitrile:acetic acid (4:1, v/v). The entire reaction mixture was loaded immediately onto a TLC plate (layer: 0.25mm, SIL G-25; DC-Fertigplatten). After air-drying, the plate was subsequently developed in a solvent system of chloroform:methanol:acetic acid:water (85:15:10:3.5, v/v/v/v). Radiolabelled products, LPA or MAG, were identified by co-migration with 16:0-LPA and 16:0-MAG standards, scraped from the TLC plate, and subjected to scintillation counting. In order to distinguish *sn*-1 and *sn*-2 DCA-MAGs, GPAT assays were carried out with 16:0 DCA-CoA as the acyl donor. The quenched GPAT assay mixture was directly subjected to boric acid-TLC with the synthesized 16:0 DCA *sn*-1 and *sn*-2 MAGs as standards. The TLC plate was developed in a solvent system containing chloroform:acetone (1:1, v/v) (Yang *et al.* 2010).

### SYBR-green qRT-PCR

Total RNA was extracted from different *B. napus* samples using an RNeasy Plant Mini Kit (Qiagen, Valencia, CA, USA) followed by DNase treatment (Qiagen). First-strand cDNA synthesis was performed in a 20 μl reaction system with 1 μg of total RNA using the QuantiTect Reverse Transcription Kit (Qiagen). The 20 μl cDNA product was diluted to 500 μl, and 2.5 μl were used in each qRT-PCR. The conditions for qRT-PCR were the same as described in Chen *et al.* (2011*b*). Three biological replicates were performed for each tested tissue/organ. UBC21 and TIP41 were used as reference genes. Each qRT-PCR resulted in a single dissociation (melting) curve. The primer sequences used in this experiment are listed in Supplementary Table S2 at *JXB* online.

### RNAi silencing of *GPAT4* in *B. napus*

A 250bp *BnGPAT4* fragment, which has no significant similarity to all other genes in the *Arabidopsis* and *B. napus* sequence database, was selected to generate the RNAi construct. An *Xho*I–*Xba*I cassette comprised of two identical 250bp PCR fragments of *BnGPAT4* was subcloned from the pHannibal vector (Helliwell and Waterhouse, 2003) into a binary vector RD400 with a napin promoter and a NOS terminator. The sequences of the primers and RNAi construct used here are listed in Table S2 at *JXB* online. The resulting RNAi construct was transformed into *Agrobacterium* strain GV3101::pMP90 (Koncz and Schell, 1986) using electroporation, according to the protocol of Weigel and Glazebrook (2006). *Brassica napus* plants (DH12075) were transformed using the *Agrobacterium*-mediated method as described in Chen *et al* (2011*b*). T_2_ transgenic *B. napus* plants were screened by germinating the seeds on MS medium (Murashige and Skoog, 1962) containing 250mg l^–1^ kanamycin. Genomic DNA PCR was performed to confirm the presence of the transgene cassette.

### Aniline blue staining of pollen tube growth in *B. napus* pistil

Hand-pollinated pistils were fixed with acetic acid/EtOH (1:3) solution overnight at room temperature. The fixed pistils were subsequently hydrated with 70, 50, and 30% ethanol and water for 10min at room temperature. The pistils were submerged in 8M NaOH overnight for softening, and then washed gently in water 3–5 times the next morning. The pistils were further treated with decolorized 0.1% (w/v) aniline blue solution containing 0.1M K_3_PO_4_ at pH 11 and 2% glycerol. The samples were then placed in the dark at room temperature overnight, following which the pistils were observed under a fluorescence microscope (Leica DMRXA) with a UV excitation source and DAPI (4′,6-diamidino-2-phenylindole) emission filter.

### Light microscopy and transmission electron microscopy

Mature seeds were enclosed in a piece of wet filter paper at 4 °C overnight. The cotyledons were cut into 1–2×1–2mm pieces, which were fixed in 0.1M sodium cacodylate buffer, pH 7.2, with 2% paraformaldehyde and 3% glutaraldehyde for 24h. The samples were washed with 0.1M sodium cacodylate buffer to remove fixative and post-fixed with 1% osmium tetroxide in 0.1M sodium cacodylate buffer for 3h. The samples were washed three times with 0.1M sodium cacodylate buffer after the post-fixing and dehydrated in a graded ethanol series (50, 70, 90, and 100% ethanol). The samples were then treated with a graded ethanol/propylene oxide series (2:1, 1:1, and 1:2, v/v), infiltrated into a propylene oxide/Spurr resin mixture (1:1, v/v) overnight and embedded in 100% Spurr resin for 24h. The samples were sectioned using an ultramicrotome. Sections of ~200nm thickness were placed onto glass slides and observed under light microscopy with a Leica DMRXA microscope coupled with a Nikon DXM1200 camera. Sections of 70–90nm thickness were placed onto 300 mesh copper grids, and stained with 4% uranyl acetate in 50% ethanol for 30min and then with Reynold’s lead citrate solution for 8min. The treated sections were observed under a Morgagni 268 (Philips-FEI) transmission electron microscope (TEM) at 80kV accelerating voltage.

### Seed oil extraction and analysis

Seed samples were boiled in 1ml of isopropanol for 10min at 80 °C. After cooling to room temperature, the samples were ground with a homogenizer in 1ml of hexane and 2ml of 3:2 hexane/isopropanol (HIP; v/v), containing 1mg ml^–1^ tri-17:0-triacylglycerol (TAG) as a standard. After centrifugation at 1500 *g*, the upper hexane phase of the mixture was extracted, and 2ml of 7:2 (v/v) HIP were added to the remaining mixture for a second extraction. The hexane extracts were combined, evaporated under nitrogen, and then a portion of the resulting extract was incubated with 1ml of methanolic HCl for 1h at 80 °C to prepare the fatty acid methyl esters.

Fatty acid methyl esters were extracted twice with 1ml of hexane and resuspended in iso-octane for GC/MS analysis, which was performed with an Agilent 6890N gas chromatograph with an Agilent 5975 Inert Mass Selective Detector. Chromatographic separation was achieved using a capillary DB-23 column (30 m×0.25 mm×0.25 μm) with a constant helium flow rate of 1.2ml min^–1^ and with temperature programmed from 90 °C to 180 °C at 10 °C min^–1^. The inlet was operated in splitless mode at 290 °C. For mass spectra detection, the solvent delay was 4min and ionization energy was 70eV; a scan mode range of 30–350 amu was used for data acquisition.

## Results

### Expression patterns of the *GPAT* family members of *B. napus*

To obtain sequence information of the *B. napus GPAT* genes, the cDNA sequences encoding eight confirmed *Arabidopsis* membrane-bound GPAT isoforms, including three isoforms located in the mitochondria (AtGPAT1–AtGPAT3) (Zheng *et al.*, 2003) and five isoforms located in the ER (AtGPAT4–AtGPAT8) (Zheng *et al.*, 2003; Li *et al.*, 2007), were used to search the *B. napus* expressed sequence tag (EST) database (Megablast, NCBI) (Zhang *et al.*, 2000) for the corresponding orthologues. Note that the putative GPAT9 was not included in the present study because a catalytic function for this category of GPAT has not been established.

As an allotetraploid closely related to *Arabidopsis*, *B. napus* has been shown to have on average six copies of the *Arabidopsis* conserved genome segments with a number of exceptions of less or more than six copies, due to genome-wide rearrangement after polyploidy (Parkin *et al.*, 2005). In the present study, the EST sequence analysis also revealed that each *Arabidopsis GPAT* gene had multiple orthologues (sequence identity >90%) in the *B. napus* genome, with the exception of *AtGPAT7* and *AtGPAT8*. In the case of *AtGPAT7*, no *B. napus* EST sequences with a significant identity were identified. For *AtGPAT8*, which has a cDNA sequence identity of >80% to *AtGPAT4*, the corresponding *B. napus* orthologues are identical to those found for *AtGPAT4* (Chen *et al.*, 2011*b*). Thus, six clusters of ESTs were identified and designated *BnGPAT1*, *2*, *3*, *4*, *5*, and *6*, which are orthologous to *AtGPAT1*–*AtGPAT6*, respectively.

**Fig. 1.**
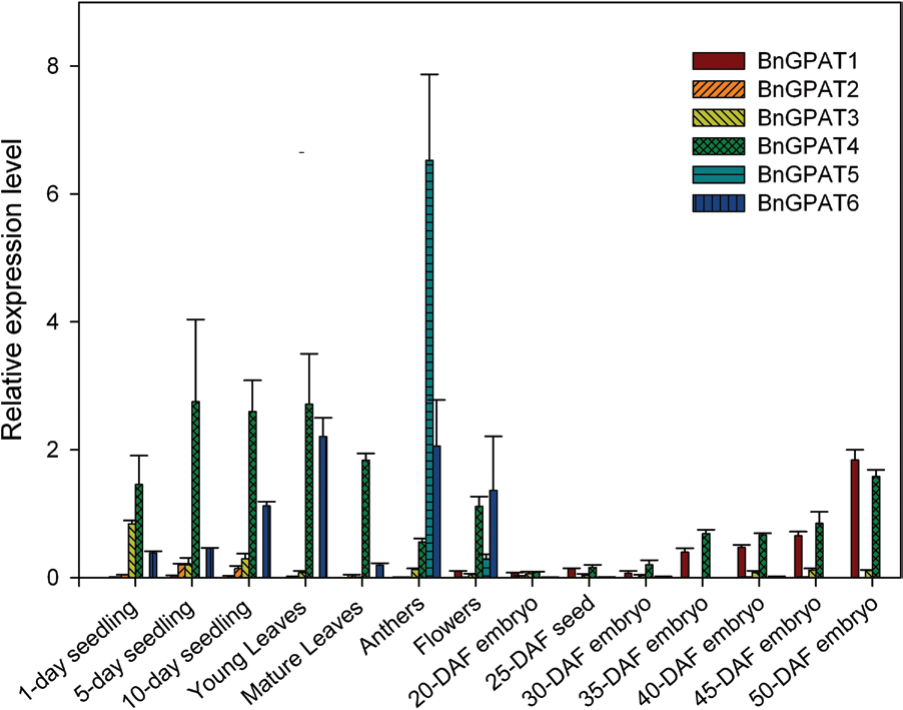
Expression patterns of *BnGPAT* genes. The transcript abundance of individual *BnGPAT* genes was evaluated in different tissues and organs using qRT-PCR. DAF, days after flowering. Error bars denote the SE of three biological replicates.

SYBR-green qRT-PCR was used to investigate the transcript abundance of the *BnGPAT* genes in different tissues and organs of *B. napus*. To simplify the qRT-PCR experiment and to have a robust comparison of the expression patterns between different *BnGPAT* genes, a pair of primers was designed based on the highly conserved regions of individual *BnGPAT* EST clusters, such that the overall transcript abundance of each *BnGPAT* orthologous cluster (i.e. *BnGPAT1–BnGPAT6*) could be obtained. As shown in Fig. 1, the expression levels of *BnGPAT1–BnGPAT6* were investigated not only in maturing embryos, but also in several other plant tissues/organs, including seedlings, leaves, dehiscent anthers, and flowers. In maturing embryos, *BnGPAT1* and *BnGPAT4* appeared to be more transcriptionally active than the other *BnGPAT* genes, suggesting a potentially important role for these isoforms during embryo development. In contrast, *BnGPAT2* and *BnGPAT3* exhibited very low transcript abundance in all investigated samples. *BnGPAT5* appeared to be expressed specifically in the anthers at a high level. *BnGPAT6* was expressed exclusively in vegetative tissues and flower organs.

The gene expression profiles suggested that *BnGPAT1* and *BnGPAT4* were the most active genes within the *GPAT* family during seed development. A previous study showed that GPAT1 deficiency in *Arabidopsis* caused reduced seed set and altered fatty acid composition in the seed oil (Zheng *et al.*, 2003). In the following study, the focus was on the enzymatic and physiological characterization of the three BnGPAT4 isoforms to gain insight into their roles in reproductive organ and seed development.

### Three BnGPAT4 isoforms exhibited *sn*-2 acyltransferase and phosphatase activities

**Fig. 2.**
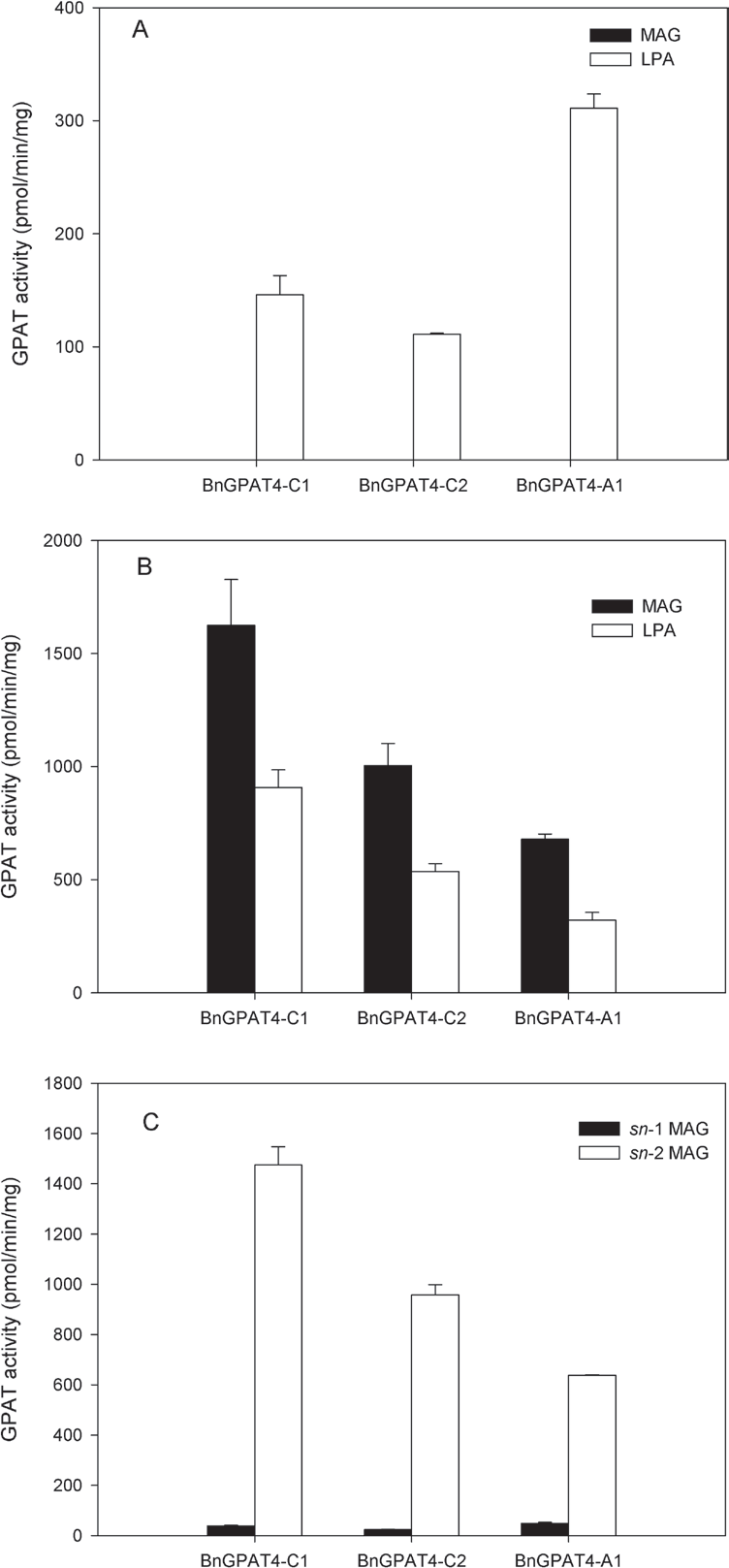
Analysis of substrate specificity and product regiospecificity of BnGPAT4 enzyme assays. (A) Enzyme activities of three BnGPAT4 isoforms using 16:0-CoA as acyl donor. (B) Enzyme activities of three BnGPAT4 isoforms using 16:0 DCA-CoA as acyl donor. (C) Regiospecificities of the 16:0 DCA-MAGs produced in BnGPAT4 enzyme assays. The enzyme assays were performed using 20 μg of yeast microsomal protein incubated with 0.5mM [^14^C(U)]glycerol 3-phosphate (0.1 μCi) and 45 μM acyl-CoA in a buffer containing 37.5mM TRIS-HCl (pH 7.5), 2mM MgCl_2_, 4mM NaF, 1mM DTT, and 0.1% BSA (w/v) at room temperature for 10min.

In *Arabidopsis*, GPAT4 possesses both *sn*-1 and *sn*-2 acyltransferase and phosphatase activities (Yang *et al.*, 2010). To investigate the catalytic functions of BnGPAT4s, *in vitro* enzyme assays were performed with individual recombinant BnGPAT4 isoforms. The expression levels of individual BnGPAT4 isoforms were first checked by western blotting to confirm that all three isoforms accumulated at a similar level per milligram of yeast crude microsomal protein (Supplementary Fig. S1 at *JXB* online). Crude microsomal fractions of yeast expressing individual BnGPAT4 isoforms were assayed with [^14^C(U)]G3P and palmitoyl (16:0)-CoA or 16:0 DCA-CoA. As shown in Fig. 2A, when the three BnGPAT4 isoforms were assayed with 16:0-CoA as acyl donor, the product was only LPA, with BnGPAT4-A1 exhibiting the highest preference for 16:0-CoA. In contrast, when 16:0 DCA-CoA was used as the acyl donor, both LPA and MAG were formed, with BnGPAT4-C1 exhibiting the highest preference for 16:0 DCA-CoA (Fig. 2B). The DCA-CoA-fuelled reactions, however, proceeded at substantially higher rates than for reactions using 16:0-CoA. In addition, acylation with DCA-CoA was required to activate the phosphatase activity of the GPAT4 isoforms. Further study on the regiospecificity of the DCA-MAGs indicated that the majority of MAG formed during the enzyme assay was *sn*-2 DCA-MAG (Fig. 2C).

### Down-regulation of *GPAT4* in *B. napus*

**Fig. 3.**
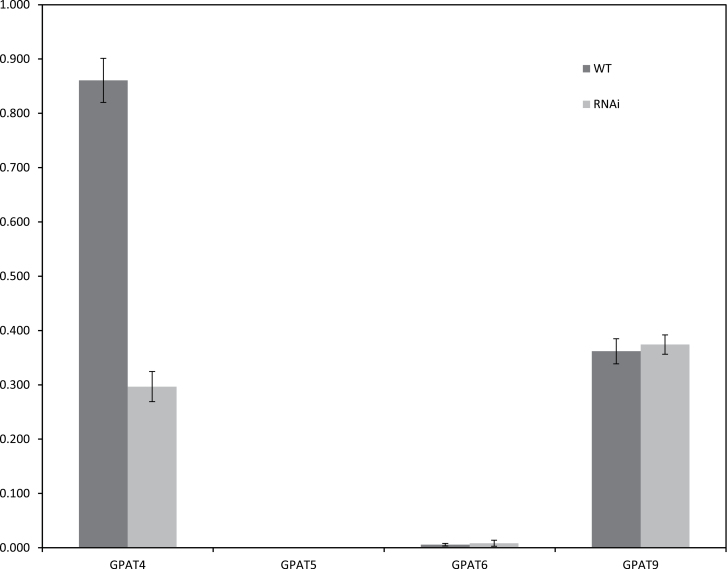
Transcript abundance of several *GPAT* family members in *gpat4* RNAi lines and the wild type (WT) of *B. napus*. qRT-PCR was performed to compare the overall transcript abundance of *BnGPAT4*, *5*, *6*, and *9* homologues in *gpat4* and WT lines. Compared with the WT line, only the transcription level of *BnGPAT4* was significantly decreased (*P*<0.05). *n*=3. Error bars denote the SE.

A previous study has shown that all three *BnGPAT4* homologues [>93% identical in open reading frame (ORF) sequences] were expressed during the embryo maturation process, although with different transcript abundance (Chen *et al.*, 2011*b*). Given that the BnGPAT4 isoforms possess similar catalytic functions, down-regulation of a single *BnGPAT4* homologue may not give a detectable phenotype due to the high probability of functional overlap. Thus, to understand the physiological functions of *BnGPAT4* genes with a robust approach, an RNAi construct was designed to down-regulate all three *BnGPAT4* genes in an embryo-specific fashion. In total, 11 T_1_ transgenic lines (confirmed by genomic DNA PCR) were generated, and five T_2_ transgenic lines were studied in detail. SYBR-green qRT-PCR was used to analyse the overall transcript abundance of *BnGPAT4* genes and several *BnGPAT* genes (that encode the ER-bound isoforms) in the RNAi lines using a mixture of developing embryos of the T_2_ transgenic lines at 40 days after flowering (DAF). As shown in Fig. 3, the expression level of *BnGPAT4* homologues was down-regulated by ~65%. Other *BnGPAT* genes, including *BnGPAT6* that has the highest sequence identity (~60%) to *BnGPAT4* within the *GPAT* family, did not change much in expression. This result confirmed that the RNAi construct was specific only to *BnGPAT4* homologues.

### *Brassica napus gpat4* lines exhibited abnormal inflorescence and reduced seed set

**Fig. 4.**
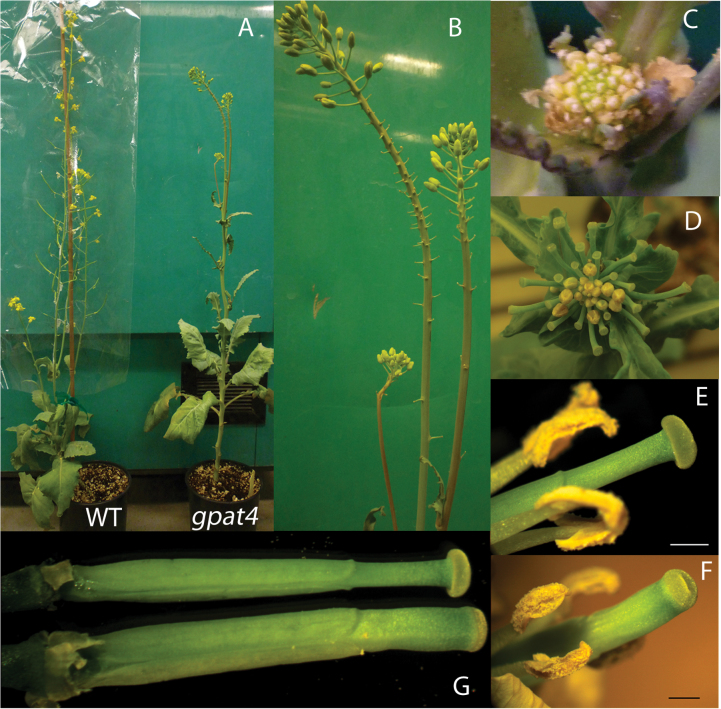
Abnormal inflorescence development in *gpat4* RNAi lines. (A) Comparison of inflorescence between a wild-type plant and a *gpat4* RNAi line. (B) Close-up view of the *gpat4* inflorescence. The development of the floral buds on the lower portion of the inflorescence was aborted in the *gpat4* lines. (C) Close-up view of *gpat4* axillary inflorescence primordia. (D) Close-up view of aborted flower buds in a later developmental stage of the axillary inflorescence. A similar phenotype was observed in all *gpat4* RNAi lines. (E–G) Comparison of the pistils of the wild type (E and upper G) and *gpat4* (F, lower G). Scale bar=1mm.

Inflorescence development was affected in *gpat4* RNAi transgenic lines. In all *gpat4* RNAi T_1_ and T_2_ transgenic lines, the development of floral buds was partially aborted, particularly on the lower portion of the inflorescence (Fig. 4A, B). Additionally, the development of axillary inflorescence primordia was also severely affected (Fig. 4C, D). Consequently, *gpat4* RNAi lines had fewer flowers in comparison with the wild-type plants. Notably, *BnGPAT4* was shown to be highly expressed in the inflorescence primordia in a previous study (Chen *et al.*, 2011*b*). These lines of evidence strongly suggest that *BnGPAT4* is pivotal for inflorescence development in *B. napus*. Additionally, the stigma and style of *gpat4* lines also appeared to be larger than those of the wild type (Fig. 4E–G).

**Fig. 5.**
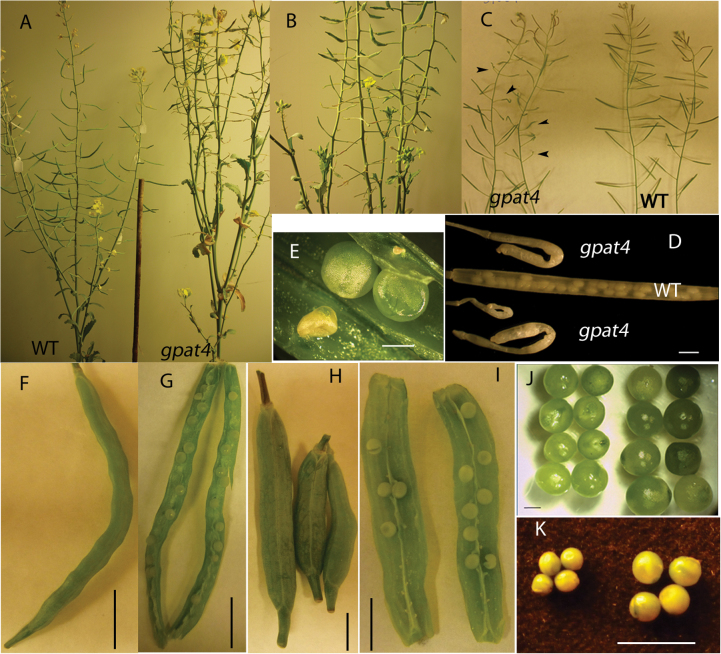
Aborted development of seeds in *gpat4* RNAi lines. (A) Comparison between a wild-type plant and a *gpat4* line during silique development. A reduced number of siliques was observed in the *gpat4* lines. (B) Close-up view of the developing siliques of a *gpat4* line. (C, D) Comparison of the developing siliques from *Arabidopsis* wild-type and *gpat4 gpat8* lines. Black arrows in (C) indicate the aborted siliques. Scale bar in (D), 1mm. (E) Seeds inside a developing *gpat4* silique. Scale bar=1mm. (F–I) Before and after opening of the developing siliques of the wild type (F, G) and *gpat4* lines (H, I). Scale bars in (F, G), 1cm. Scale bars in (H, I), 0.5cm. (J, K) Comparison of the 10 days after flowering (DAF; J) and 25 DAF (K) developing seeds of the wild type (left) and *gpat4* lines (right). Scale bar in (J), 1mm. Scale bar in (K), 1cm.

The *gpat4* RNAi lines also exhibited reduced seed yield. In the T_1_ generation, a few independent *gpat4* RNAi lines were completely sterile. The majority of *gpat4* RNAi lines had short and stocky siliques with <10 seeds within each silique (Fig. 5B, E, H, I), while the wild-type siliques were much longer, containing on average >20 seeds per silique (Fig. 5A, F, G). The *B. napus* *gpat4* seeds also appeared to be larger (Fig. 5J, K) and had significantly increased weight (*P*<0.001) compared with wild-type seeds (Table 1). In *Arabidopsis gpat4 gpat8* double T-DNA lines, reduced seed set was also observed, but it was not as severe as in the *B. napus gpat4* lines (Fig. 5C, D).

**Table 1.** Seed oil analysis of the wild-type and *gpat4* lines

	Weight per 10 seeds (mg)	Oil content wt%	Fatty acid composition (mol%)							*n*
			16:0	18:0	18:1	18:2	18:3	20:0	20:1	
Average	38.025	32.3	4.626	1.960	61.360	17.707	10.830	0.405	0.667	3
SE	0.512	1.3	0.039	0.047	0.446	0.281	0.238	0.047	0.025	
Average	38.150	34.4	4.830	1.918	61.225	17.392	11.102	0.507	0.780	4
SE	0.330	0.9	0.049	0.034	0.234	0.074	0.138	0.027	0.007	
Average	47.900	27.8	5.133	1.441	51.240	24.148	14.512	0.330	0.847	3
SE	1.421	0.1	0.047	0.032	0.618	0.488	0.117	0.051	0.020	
Average	48.825	26.4	5.299	1.308	51.497	22.774	15.365	0.356	0.835	4
SE	1.477	0.9	0.093	0.029	0.783	0.375	0.289	0.029	0.016	
Average	50.000	24.5	5.057	1.318	51.986	22.124	15.842	0.358	0.859	4
SE	0.908	1.1	0.107	0.029	0.909	0.583	0.355	0.031	0.011	
Average	47.025	26.8	4.909	1.521	57.679	20.555	11.899	0.391	0.837	4
SE	1.765	1.4	0.092	0.037	0.899	0.723	0.248	0.021	0.021	
Average	48.150	28.3	4.650	1.269	58.744	19.479	12.306	0.327	0.932	4
SE	0.681	1.1	0.062	0.027	0.844	0.334	0.630	0.025	0.034	

### Down-regulation of *GPAT4* affected pollen development in *B. napus*

**Fig. 6.**
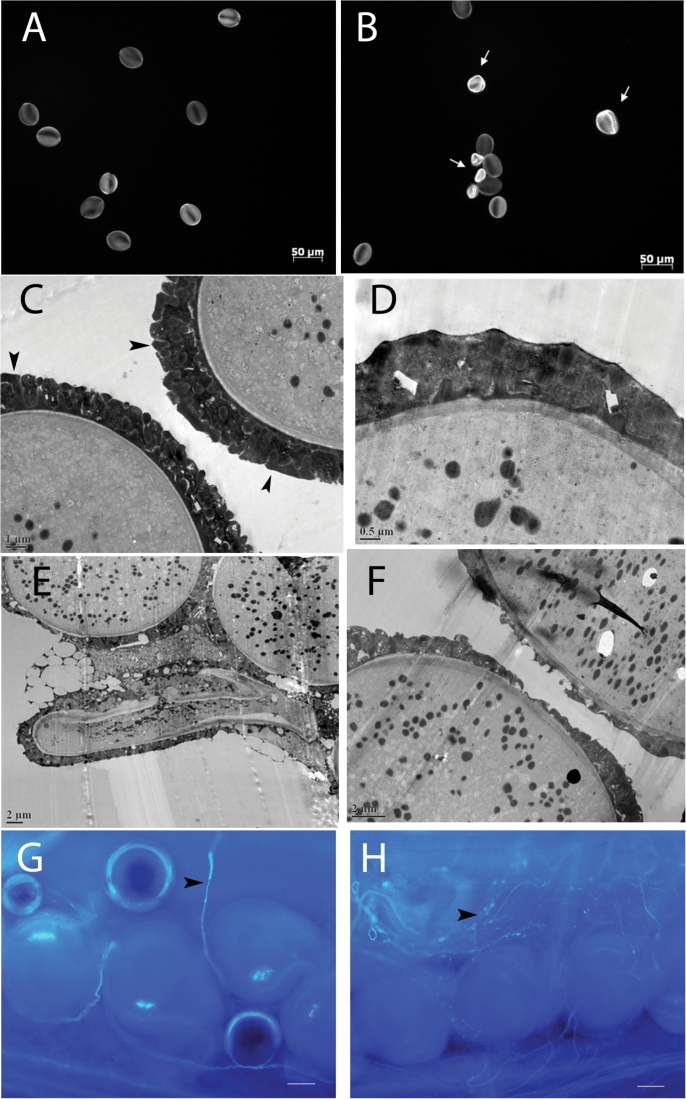
Abnormal pollen grains of the *gpat4* RNAi lines. (A, B) Pollen grains of the wild type (A) and *gpat4* (B) lines under light microscopy. White arrows indicate the deformed pollen grains. (C) Transmission electron microscopy (TEM) image of the wild-type pollen grains. Black arrows indicate the exine deposited on the pollen coat. (D–F) TEM images of the *gpat4* pollen grains. Reduced deposition of exine was observed in some of the *gpat4* pollen grains. (G, H) Aniline blue staining showing the *in vivo* pollen tube growth (as indicated by black arrows) inside the pistils of the self-pollinated wild type (G) and *gpat4* (H) lines. Scale bars in (G, H), 0.1mm.

To investigate the cause(s) of the reduced seed yield of the *B. napus gpat4* lines, the mature pollen grains of the *gpat4* lines were first examined under light microscopy and TEM. As shown in Fig. 6B, under light microscopy, some of the *gpat4* pollen grains were deformed or collapsed. Further examination under TEM revealed that in addition to the deformed pollen grains, some of the normal-shaped *gpat4* pollen grains had defective exine deposition on the pollen wall (Fig. 6C–F). Considering that *B. napus* flowers produce an excessive amount of pollen grains to ensure sufficient fertilization, it is possible that the remaining *gpat4* pollen grains of normal appearance could enable normal pollination. Thus, the *in vivo* pollen tube growth of the *gpat4* lines was examined. Both wild-type and *gpat4* pistils were hand pollinated with their own pollen grains, and then stained with aniline blue at 2 DAF. As shown in Fig. 6G, H, in both wild-type and *gpat4* lines, the germinated pollen tubes could reach the ovary locules. Thus, it appeared that although a portion of pollen grains were adversely affected, the remaining viable pollen grains of the *gpat4* lines had the ability to germinate *in vivo*.

### Impaired female fertility reduced seed yield in *B. napus* *gpat4* lines

**Fig. 7.**
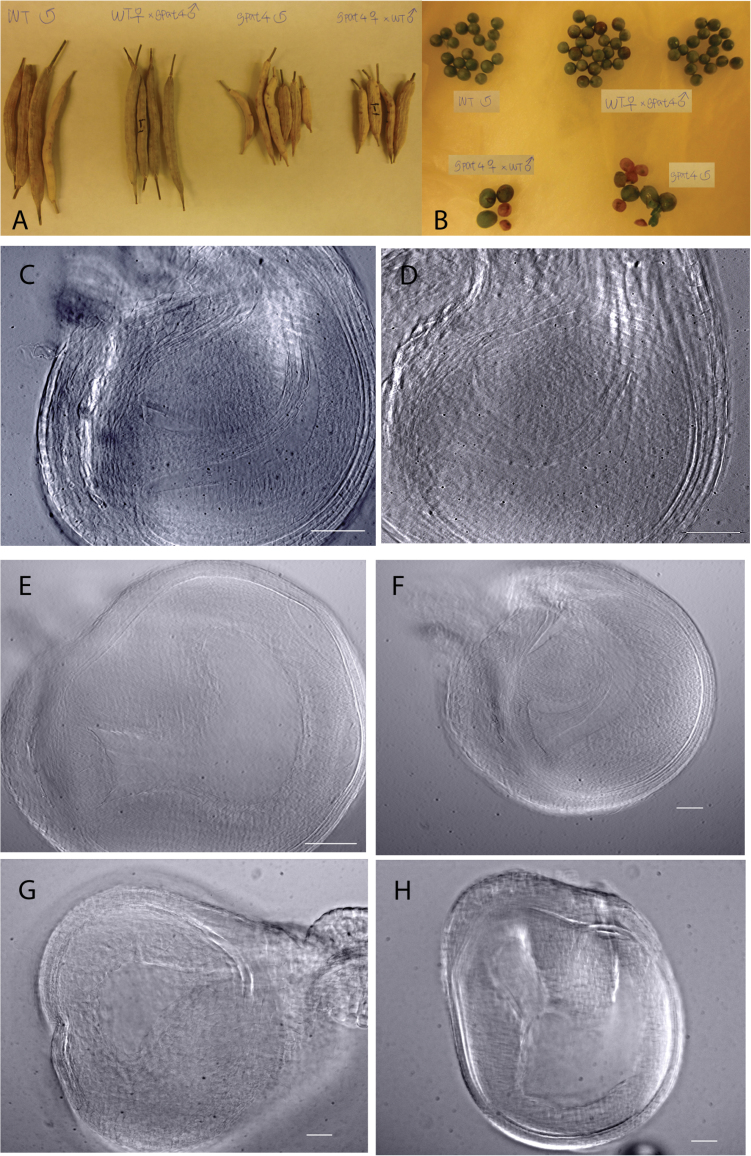
Reciprocal cross result and examination of the embryo sacs of *gpat4*. (A, B) Siliques and seeds from reciprocal crosses between wild-type and *gpat4* plants. Siliques and seeds from wild-type plants that were pollinated with *gpat4* pollen grains were as normal as those from the self-pollinated wild-type plants. Pollination of *gpat4* plants with wild-type pollen grains resulted in fewer seeds per silique, similar to the self-pollinated *gpat4* plants. (C, D) Unfertilized wild-type (C) and *gpat4* (D) ovules collected on the first day of stamen adhesion. The embryo sacs of the wild type and *gpat4* exhibited no morphological difference at this stage. Scale bars=0.1mm. (E) A wild-type ovule at 4 d post-pollination. Scale bar=0.1mm. (F–H) Ovules of *gpat4* at 4 d post-pollination. The embryo sac inside the wild-type ovule was different from that of *gpat4*. Scale bars=0.1mm.

To understand further the reason for the reduced seed yield of the *B. napus gpat4* lines, reciprocal crosses were performed between the *gpat4* and wild-type *B. napus* lines. As shown in Fig. 7A, B and Supplementary Table S1 at *JXB* online, seed development and seed number per silique were normal when wild-type stigmas were pollinated with *gpat4* pollen grains. On the other hand, pollination of *gpat4* flowers with wild-type pollen grains resulted in fewer seeds per silique, as in the self-pollinated *gpat4* plants. These results suggested that the deficiency of *GPAT4* in *B. napus* severely affected female fertility, and the remaining viable *gpat4* pollen grains were capable of normal fertilization.

The developing embryo sac of the *gpat4* lines was examined further before and after pollination. Unpollinated and pollinated pistils were collected on the first and fourth day of anther dehiscence, respectively. Although it was difficult to distinguish the individual nuclei, the *gpat4* embryo sacs exhibited no apparent morphological difference compared with the wild type before pollination (Fig. 7C, D). At 4 DAF, however, most of the *gpat4* embryo sacs exhibited arrested development (Fig. 7F) or deformed shapes (Fig. 7G, H). These results indicated that the aborted seed development of *gpat4* may be caused by defective embryo sac development after pollination.

### *Brassica napus gpat4* mature embryos exhibited alterations in cutin content and monomer profile

**Fig. 8.**
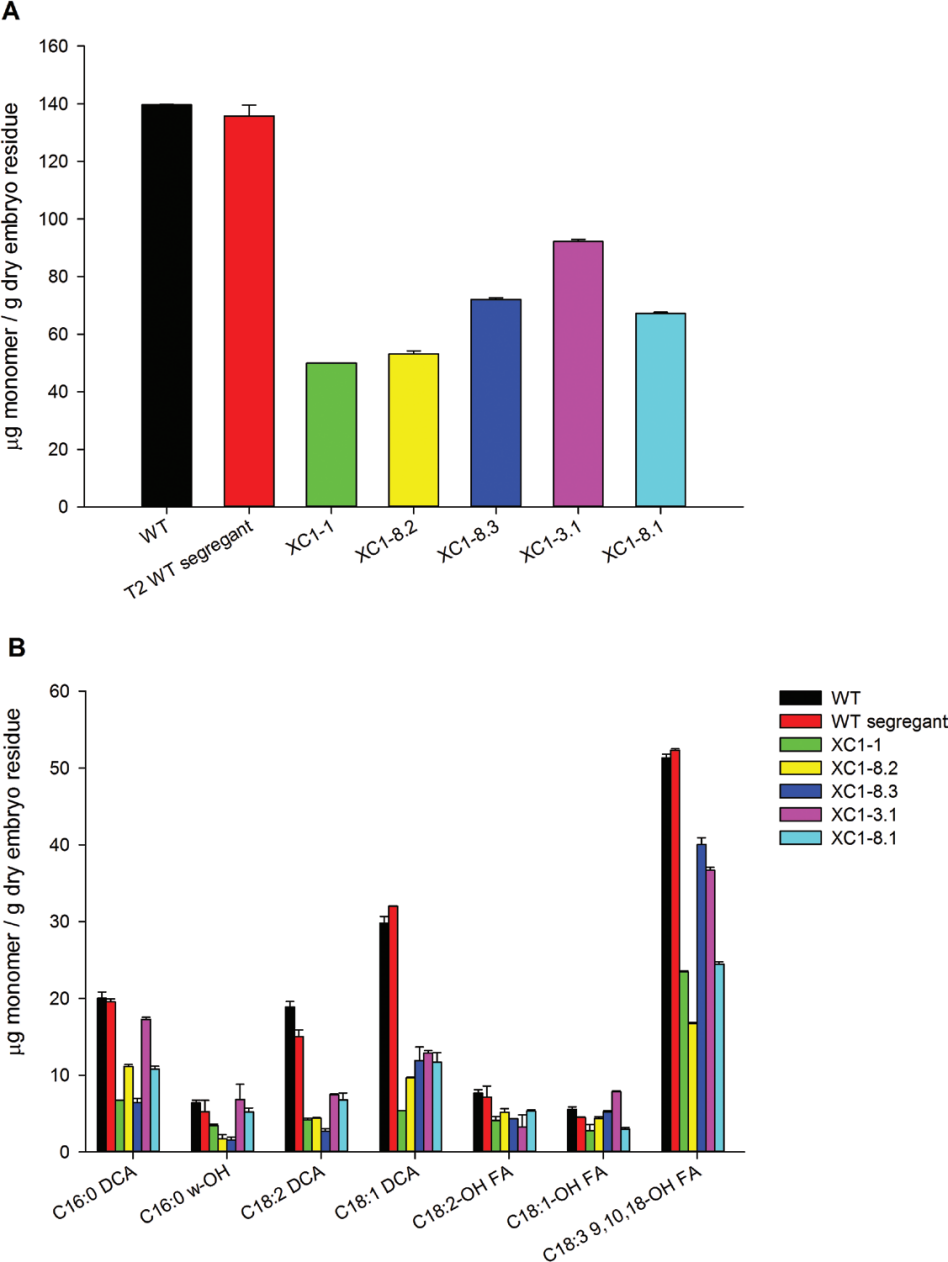
Down-regulation of *GPAT4* homologues resulted in decreased cutin monomer load. (A) The *gpat4* lines had decreased cutin content by >30% (*P*<0.05) in the mature embryos compared with the wild-type (WT) lines; *n*=3. (B) The cutin monomer profile of the *gpat4* and WT lines; *n*=3. The error bar denotes the SD. (This figure is available in colour at *JXB* online.)

Previous studies have shown that GPAT4 is involved in biosynthesis of the cuticle layer covering the aerial tissues of the plant (Li *et al.*, 2007). Given that the embryo is also covered by a thin layer of cuticle (Molina *et al.*, 2006), it was of interest to investigate if the cutin content of the *gpat4* mutant embryos was affected. As shown in Fig. 8, the total cutin content of the mature embryos of RNAi lines was significantly decreased by >30% compared with the wild-type line. The cutin monomer profile was also changed in the RNAi lines. Among the monomers, 18:1 DCA, 18:2 DCA, and 18:3 9,10,18 hydroxy fatty acid (18:3 9,10,18-OH FA) exhibited significant reductions.

### *Brassica napus gpat4* seeds exhibited decreased oil content and altered fatty acid composition

**Fig. 9.**
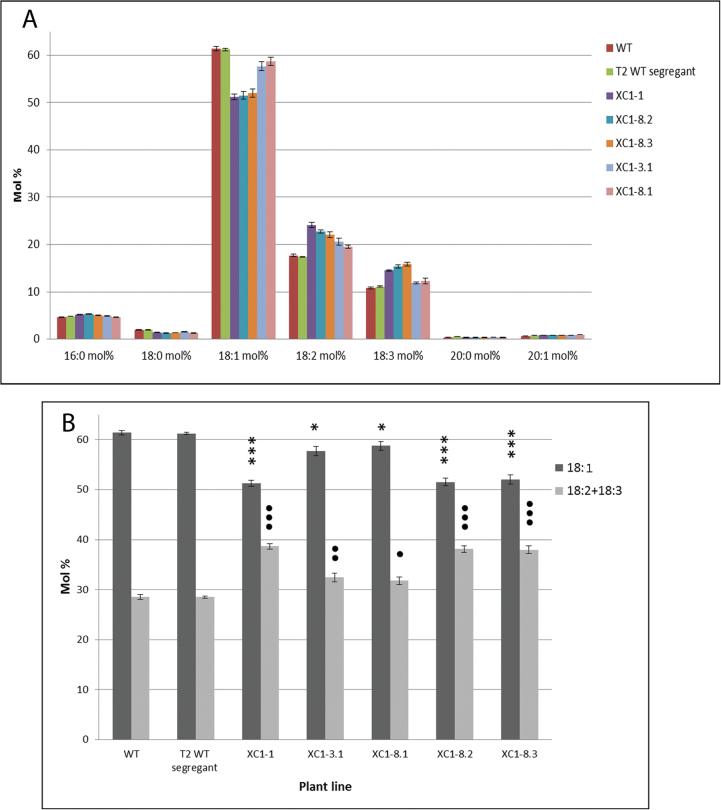
Analysis of the seed oil fatty acid profile of wild-type and T_2_ *gpat4* lines. (A) Relative mole fraction (mol%) of fatty acids in the total seed oil. (b) A detailed chart of the mole fractions of oleic (18:1), linoleic (18:2), and α-linolenic (18:3) acids of wild-type and *gpat4* lines. Asterisks (*) indicate the significant differences in 18:1 contents between the wild type and the *gpat4* lines as determined by *t*-test. Black circles indicate significant differences in the total contents of 18:2 and α-18:3 between the wild type and *gpat4* lines as determined by *t*-test. One asterisk or black circle indicates *P*<0.05; double asterisks or black circles indicate *P*<0.01; triple asterisks or black circles indicate *P*<0.001. *n*=3 or 4. Error bars denote the SD.

The oil content and fatty acid composition of the T_2_ generation *gpat4* seeds were also different from those of the wild type. As shown in Table 1, the seed oil contents (wt%) were significantly decreased (by 12.4–24.1% on a relative basis, *P*<0.05) in the *gpat4* lines compared with the wild type and segregant lines. Furthermore, the fatty acid profiles were also different in the *gpat4* lines. The molar proportions of oleic acid (18:1^*cis*∆9^) were decreased by 4.3–16.5% on a relative basis and the molar proportions of linoleic acid (18:2^*cis*∆9,12^) and α-linolenic acid (18:3^*cis*∆9,12,15^) were increased to varying degrees (Table 1, Fig. 9). Similar changes in fatty acid composition were also observed in the T_1_ seeds (Supplementary Fig. S2 at *JXB* online).

**Fig. 10.**
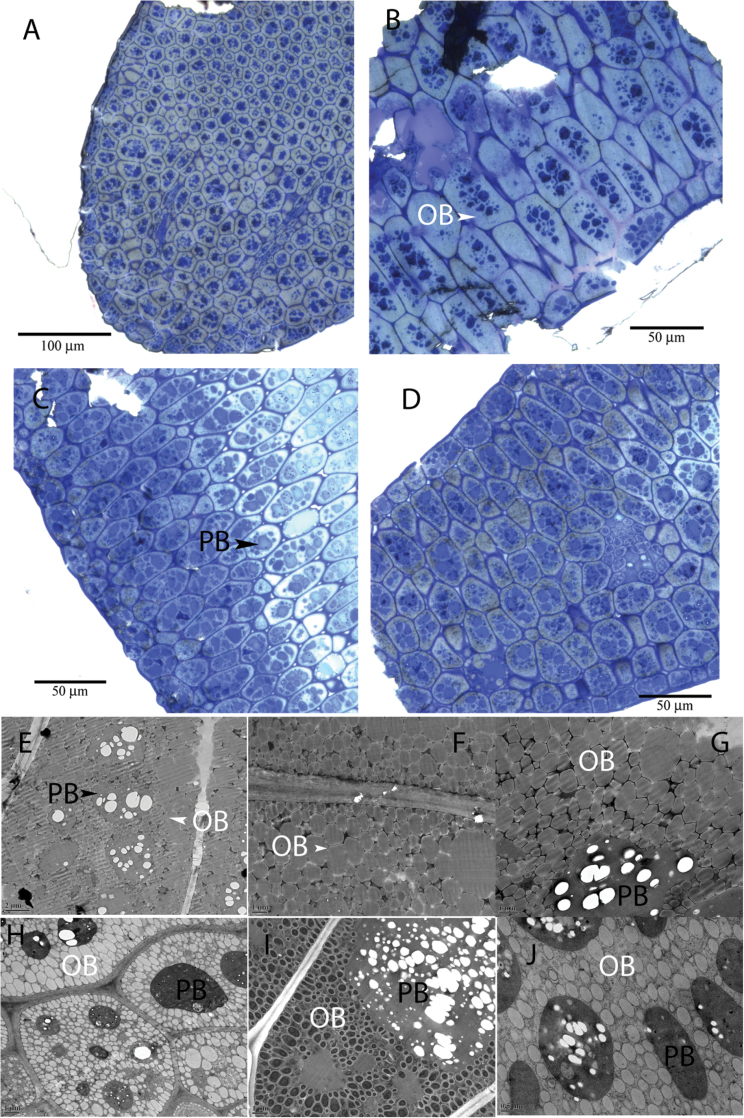
Mature embryo sections of the wild type and *gpat4*. (A, B) Sections of wild-type embryo cotyledons. (C. D) Sections of *gpat4* embryo cotyledons. More protein bodies were present in the *gpat4* cotyledon cells than in the wild-type cotyledons. (E–G) Transmission electron microscopy (TEM) images of the wild-type cotyledons. The T_2_ wild-type segregant line exhibited the same cellular morphology. (H–J) TEM images of the *gpat4* cotyledons. PB, protein body. OB, oil body. The oil bodies in wild-type cotyledon cells were compacted and connected to each other; in contrast, the oil bodies in *gpat4* cotyledon cells were disconnected from each other and appeared to be smaller and rounder than those observed in the wild type.

To investigate the physiological effect of *GPAT4* down-regulation on oil body accumulation, cotyledon sections of mature embryos of *gpat4* and the wild type were examined under light microscopy and TEM. In wild-type mature embryo cells, the oil bodies were present in the periphery of the cells and between the centrally located protein bodies. As shown in Fig. 10A–D, the protein bodies appear as dark blue spheres, surrounded by the oil bodies (light blue or light grey colour) within each cell. There appeared to be more protein bodies present in the *gpat4* embryo cells (Fig. 10C, D) than in the wild type (Fig. 10A, B). The ultrastructure of the oil bodies was also different between the wild and *gpat4* lines as observed under TEM. The oil bodies in the wild-type embryo cells were pressed against each other into irregular shapes (Fig. 10E–G). In contrast, oil bodies within the *gpat4* embryo cells were rounder, smaller, and often disconnected from each other (Fig. 10H–J).

## Discussion

A previous study of *gpat4* RNAi lines under the control of the CaMV35S promoter found that the only detectable phenotype was a cuticle defect on the epidermis (Chen *et al.*, 2011*b*); however, two (out of 10) T_1_ lines exhibited abnormal inflorescence development and severely reduced seed yield, the same abnormality observed in the current napin promoter-directed *gpat4* RNAi lines. It appeared that the napin promoter-directed RNAi construct was more effective in down-regulating the expression of *GPAT4* in the reproductive organs of *B. napus*. The napin promoter used in the present study originates from a *napA* gene (a member of the *napin* gene family, GenBank accession no. J02798), which encodes a 1.7S seed storage protein in *B. napus* (Josefsson *et al.*, 1987). The expression patterns of the *napin* gene family or the corresponding promoters have mainly been investigated within the developing seeds (Blundy *et al.*, 1991; Kridl *et al.*, 1991; Ellerstrom *et al.*, 1996); thus, it is not clear whether *napin* genes could also be expressed in other plant organs. Notably, the corresponding orthologous genes in *Arabidopsis* (AT4G27170, AT4G27140, AT4G27160, AT4G27150, and AT5G54740), which encode seed storage albumin proteins, are expressed not only in developing seed but also in pollen grain (Supplementary Fig. S3 at *JXB* online, via the AtGenExpress Visualization Tool; Schmid *et al.*, 2005). Beside the promoter, another factor that can affect the organ targeting of RNAi is that gene silencing regulated by RNAi can spread locally and systemically (Klahre *et al.*, 2002; Himber *et al.*, 2003; Dong *et al.*, 2011). Thus, it is possible that the RNAi construct was expressed at a higher level than the natural expression level driven by a napin promoter in the reproductive organs, such as the flower primordium and pollen grains. It was previously shown that the *BnGPAT4* homologues are expressed at the highest level in the flower primordium among all the investigated vegetative organs (Chen *et al.*, 2011*b*). Therefore, it is believed that BnGPAT4s are important for the development of flower primordium, possibly with a role in providing the polyester surface layer for this developing organ given the enzymatic activity of the corresponding enzymes. The disruption of normal flower primordium development will subsequently affect the flower organ development. As a result, a series of developmental defect were observed in the flower organs. The *B. napus gpat4* lines not only produced collapsed pollen grains but also had pollen grains with reduced deposition of exine. The development of pollen is a complex process, which is known to be closely connected to lipid metabolism within the tapetum and pollen grain itself (Zheng *et al.*, 2003; Wang *et al.*, 2008; Beaudoin *et al.*, 2009). Two previous studies in *Arabidopsis* revealed that down-regulation of two other *GPAT* family members, *AtGPAT1* and *AtGPAT6*, caused defective ER profiles in the tapetum, which further affected nutrient secretion from the tapetum to pollen grains (Zheng *et al.*, 2003; Li *et al.*, 2012). It is possible that *BnGPAT4* is similarly involved in tapetum development so as to affect pollen development.

The *gpat4* seeds not only were larger during development, but also had increased weight at maturity compared with wild-type seeds. This was possibly caused by an increased source-to-sink ratio in the RNAi lines, given that the *B. napus* *gpat4* lines had much less seed set compared with the wild type due to defective inflorescence development and reduced female fertility of the *gpat4* lines. Although the seed weight increased, the seed oil content (wt%) in the *gpat4* lines was decreased. These results indicate that suppression of *GPAT4* expression may have resulted in restricted use of cellular carbon for storage lipid biosynthesis. Furthermore, in contrast to the wild type, the oil bodies in the *gpat4* mature seeds were rounder, smaller, and disconnected from each other. Previous studies have suggested that oleosin, which is the major protein present in the phospholipid monolayer of oil bodies, plays an important role in controlling the size of oil bodies (Ross *et al.*, 1993; Tzen *et al.*, 1993; Ting *et al.*, 1996; Siloto *et al.*, 2006). These studies indicated that higher TAG to oleosin ratios resulted in larger oil bodies, while lower TAG to oleosin ratios resulted in smaller oil bodies. Thus, the smaller and rounder oil bodies in the *gpat4* seeds could be caused by a lower TAG to oleosin ratio imparted by the decreased oil content in the seeds.

In the present study, it was demonstrated that the BnGPAT4 isoforms are similar to AtGPAT4 in being able to use DCA-CoA as acyl substrate, and all possess *sn*-2 acyltransferase and phosphatase activities. Such enzymatic activities were suggested to be related to the role of GPAT4 in cutin biosynthesis in providing intermediates for cutin polymer assembly (Yang *et al*., 2010, 2012). Cutin, a major component of the cuticle layer, has been found on the epidermis of aerial organs and developing embryos (Molina *et al.*, 2006). As previous studies have only revealed the role of *GPAT4* in cuticle formation of vegetative tissues (Li *et al.*, 2007; Chen *et al.*, 2011*b*), in the present study, it was further demonstrated that down-regulation of *BnGPAT4* homologues also affected embryo cutin biosynthesis. It is not clear whether such a substantial reduction in cutin load could potentially affect embryo development. It has been reported that in an *Arabidopsis* cuticular wax mutant line *resurrection1* (*rst1*), 70% of the seeds underwent arrested embryo development. Interestingly, the surviving shrunken *rst1* seeds exhibited a similar phenotype to the *gpat4* seeds, which was characterized by reduced seed oil content and altered fatty acid composition (Chen *et al.*, 2005).

Although GPAT can catalyse the non-substituted acyl-CoA-dependent production of LPA, which is the initial step in bringing the glycerol backbone into the TAG synthesis network, there are few experimental data, so far, to demonstrate that the currently known ER GPATs are directly involved in the biosynthesis of storage lipids in plants. On the other hand, the strong *sn*-2 acyltransferase activity using ω-oxidized acyl-CoAs as substrates of GPAT4 would provide substrates for extracellular lipid polyester biosynthesis rather than feed into the Kennedy pathway (Weselake *et al.*, 2009) involved in storage lipid biosynthesis. Therefore, the changes in storage lipid of the transgenic lines would be an indirect effect of abnormal embryo development. In the enzymatic assay reported here, only LPA, but not MAG, was produced with non-substituted acyl-CoA (16:0-CoA) as the acyl donor. It would be interesting to know if this catalytic reaction with non-substituted acyl-CoA functioned *in vivo.*

In summary, the results from the present study have revealed critical physiological roles for BnGPAT4s in reproductive organ and embryo development. This information adds further to knowledge on the physiological roles of these multifunctional enzymes in plant development.

## Supplementary data

Supplementary data are available at *JXB* online.

Figure S1. Western blot of individual BnGPAT4 isoforms expressed in yeast strain *gat1Δ*.

Figure S2. Fatty acid composition analysis indicated decreased content of 18:1 and increased content of 18:2 and 18:3 in the *B. napus gpat4* T_1_ seed oil.

Figure S3. The expression patterns of *Arabidopsis* genes encoding seed storage albumin proteins.

Table S1. Seed set analysis of the reciprocal crosses between *gpat4* and wild-type *B. napus* lines.

Table S2. Sequences of primers and the RNAi construct used in the present study.

Supplementary Data
