# Supplementary material for: Glycerol-3-phosphate acyltransferase 4 is essential for the normal development of reproductive organs and the embryo in Brassica napus
Source: J Exp Bot. 2014 May 12;65(15):4201–15. doi: 10.1093/jxb/eru199 (PMC4112632; doi:10.1093/jxb/eru199)
Supplement: Supplementary Data [file supp_65_15_4201__index.html]

Supplementary Data 

# Glycerol-3-phosphate acyltransferase 4 is essential for the normal development of reproductive organs and the embryo in *Brassica napus*

## Supplementary Data

Data files

**Files in this Data Supplement:**

- Supplementary Data - Supplementary Data
- Supplementary Data - Supplementary Data
- Supplementary Data - Supplementary Data
