# Supplementary material for: Glycerol-3-phosphate acyltransferase 4 is essential for the normal development of reproductive organs and the embryo in Brassica napus
Source: J Exp Bot. 2014 May 12;65(15):4201–15. doi: 10.1093/jxb/eru199 (PMC4112632; doi:10.1093/jxb/eru199)
Supplement: Supplementary Data [file supp_eru199_jexbot118539_file003.pdf]

Fig. S1

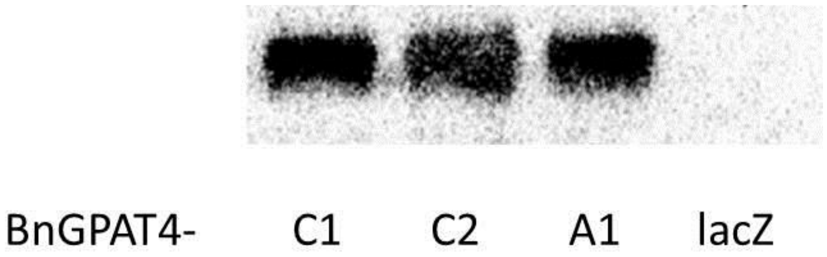

Figure S1. Western blot of individual BnGPAT4 isoforms expressed in yeast strain *gat1Δ*. C1, C2, and A1 are the three BnGPAT4 homologs BnGPAT4-C1, BnGPAT4-C2 and BnGPAT4-A1, respectively. LacZ referred to the microsome sample extracted from LacZ-transformed yeast strain, which was used as a negative control (Chen et al., 2011b).

Fig. S2

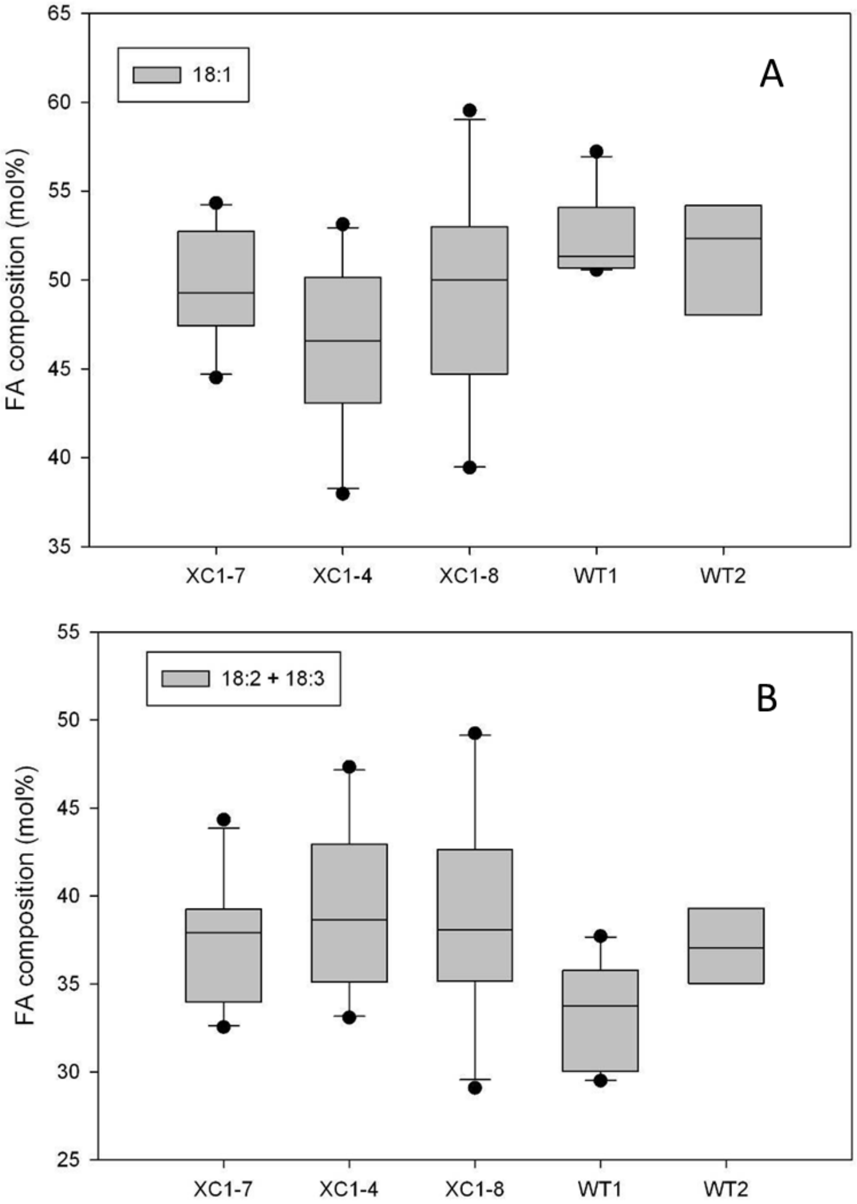

Figure S2. Fatty acid composition analysis indicated decreased content of 18:1 and increased content of 18:2 and 18:3 in the *gpat4 B. napus* T_1 seed oil.

Box plots were used to represent the content of 18:1 and the total content of 18:2 and 18:3 (18:2+18:3) of the individual seeds from each *B. napus* plant. (A) The 18:1 content and (B) the 18:2 and 18:3 content of three independent T_1 *gpat4* lines (XC1-7, XC1-4, and XC1-8) and two wild-type plants (WT1 and WT2).

Fig. S3

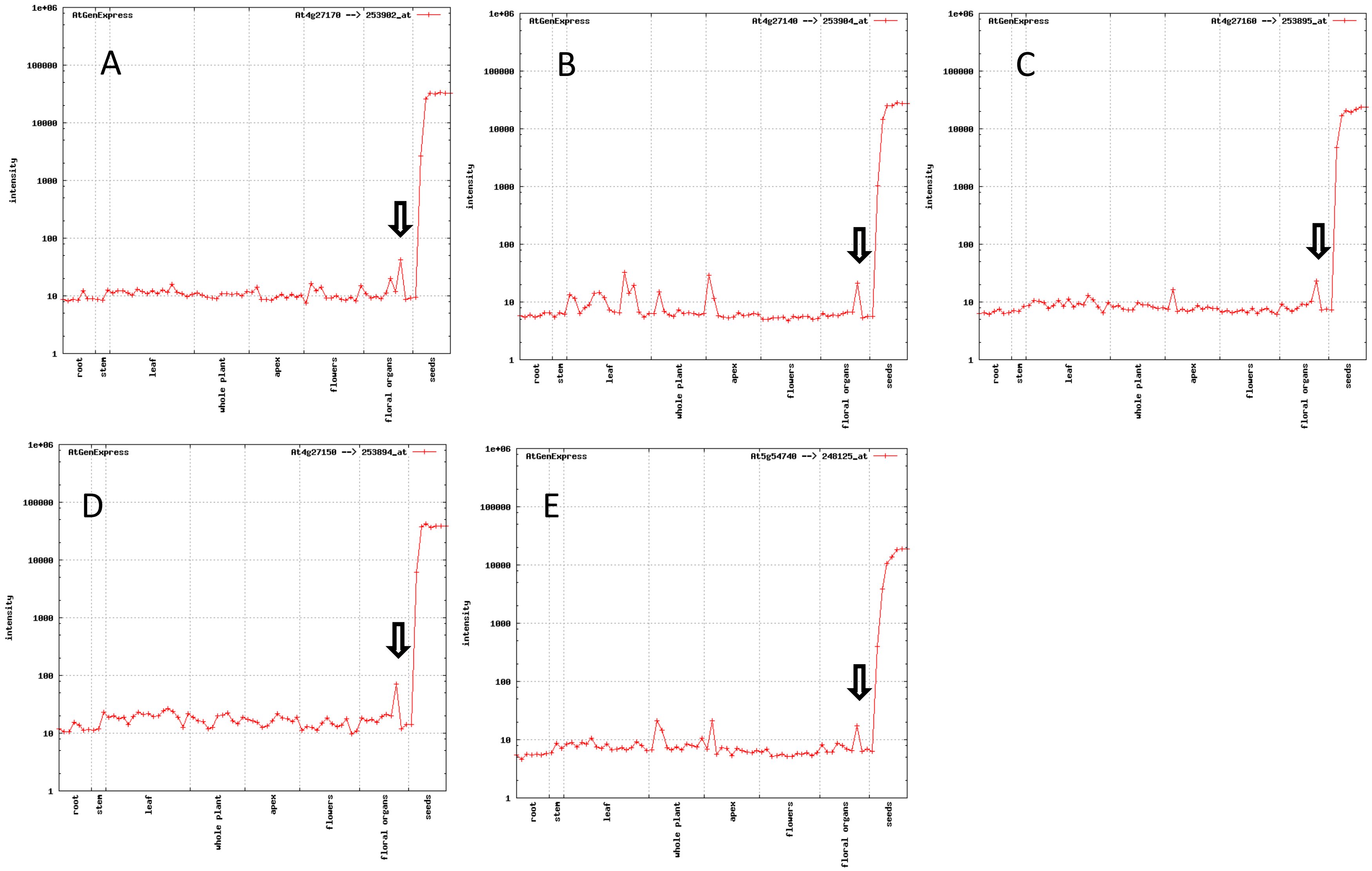
A
AtGenExpress
At4g27170 --> 253902_at
B
AtGenExpress
At4g27140 --> 253904_at
C
AtGenExpress
At4g27160 --> 253895_at
D
AtGenExpress
At4g27150 --> 253894_at
E
AtGenExpress
At5g54740 --> 248125_at
intensity
1e+06
100000
10000
1000
100
10
1
root
stem
leaf
whole plant
apex
flowers
floral organs
seeds

Figure S3. The expression patterns of Arabidopsis genes encoding seed storage albumin proteins. Data were generated using the AtGenExpress Visualization Tool with the experiment of AtGE development and avsolute values (Schmid et al., 2005). A-E represent the expression patterns of AT4G27170, AT4G27140, AT4G27160, AT4G27150 and AT5G54740, respectively.
